# Wishes, conflicts, and support needs of informal caregivers of patients in the palliative phase: A qualitative study

**DOI:** 10.1177/13591053251357769

**Published:** 2025-08-07

**Authors:** Anne Looijmans, Marrit Annika Tuinman, Marieke Nanninga, Mariët Hagedoorn

**Affiliations:** 1 University of Groningen, University Medical Center Groningen, The Netherlands; 2 Zorgbelang Groningen, The Netherlands

**Keywords:** caregiver burden, conflicts, informal care, palliative phase, support

## Abstract

This study explored wishes, conflicts, beneficial, and wished support of informal caregivers (ICGs) providing care to a palliative ill close other. We interviewed five current and 15 bereaved ICGs (25–75 years), and used thematic analyses. ICGs wished to continue life as normal as possible, spend time together and with their family, comfort their close other, and continue own activities. Caregivers experienced conflicts in balancing caregiving and own activities, quality time with each other and social contacts, the level of professional or informal support preferred, and in their shifting role from partner/child to nurse. ICGs experienced practical support, being heard, and good professional support as helpful. An overview of available support options, one contact for administrative issues, and an environment that pays attention to ICGs’ wellbeing could make caregiving easier. Formal carers and digital tools can support caregivers in balancing wishes and boundaries with the requests of caregiving to decrease conflicts.

## Introduction

The message that a close other has an incurable, life-threatening illness, is the start of an unpredictable, life-changing trajectory with a certain outcome. People close to the patient will lose their close other within a couple of months or several years after diagnosis, for example in case of advanced pancreatic cancer (Li et al., 2004) or amyotrophic lateral sclerosis (ALS; Chiò et al., 2009). Family members and friends will often provide a large part of the care the close other needs, next to formal care. These informal caregivers (ICGs) may assist their close other with personal care, symptom management, transportation, emotional and spiritual support, administrative issues, and communication with care professionals (Candy et al., 2011).

ICGs indicate that caring for a close other with an incurable, life-threatening illness is important to them and makes them feel good (Zapart et al., 2007). Caregiving is associated with positive aspects, such as spending more time together, experiencing more emotional closeness in spousal relationships, admiration, increases in personal strength, and post-traumatic growth (Flemming et al., 2020; McDonald et al., 2018; Ruf et al., 2009; Zwahlen et al., 2010). The downside of caring for a relative or friend with an incurable illness is that it is often associated with higher levels of anxiety, depression, anger, loneliness, and burden (Barg et al., 1998). Although many informal caregivers care out of love, numerous experience increasing levels of physical, psychological, and financial burden (Mangan et al., 2003).

In 2003, the view on care at the end of life shifted from a model that sharply divides curative care from terminal care, to a model in which palliative symptom management gradually increases when the amount of curative care decreases (Lynn and Adamson, 2003). Palliative care aims to “improve the quality of life of patients and their families who are facing problems associated with life-threatening illness” (World Health Organization, 2020), for example by relieving patient’s pain or difficulty in breathing. Palliative care should be initiated from the diagnosis of a life-threatening illness and be continued onward to prevent and relieve suffering whereas palliative *terminal* care can be initiated when the patient’s life expectancy is estimated to be shorter than 3 months (Distelmans and Bauwens, 2008). Starting palliative care early is beneficial for patients’ quality of life (Qureshi et al., 2019), in avoiding unnecessary care in the last phase of life (Boddaert et al., 2022), prolonging survival (Temel et al., 2010), and for improving of caregivers’ levels of depression (Alam et al., 2020) and satisfaction with care (McDonald et al., 2017).

To adequately support ICGs from the moment their close other is diagnosed with an incurable, life-threatening illness onward, it is important to gain insight into their experiences and support needs during the course of their caregiving. A systematic review showed that palliative home-based patients’ and their carers’ most frequently reported unmet need was effective communication with and among health professionals for better continuation of care, and that they missed attention for their spiritual and psychological needs (Ventura et al., 2014). Some studies suggest that ICGs experienced difficulties in balancing caregiving responsibilities with their own needs, and prioritized the care for and well-being of the close other over their own well-being (de Wit et al., 2019; Grande et al., 1997; Zapart et al., 2007). When people cross their boundaries or compromise their own needs in favor of others, this may lead to conflicts within themselves or with other persons (Barki and Hartwick, 2004; Drory and Ritov, 1997). These conflicts with others have been found to be a strong contributor to daily stress (Bolger et al., 1989). As the amount and the complexity of the care that is needed will further increase when the close other’s disease progresses, it is likely that these conflicts within themselves and with others will also intensify over time, adding to the stress of caregivers (Alam et al., 2020).

Therefore, to understand how to support ICGs to provide care in a way that preserves their own well-being during the palliative months or years, we need to gain more insight into what ICGs wish, the boundaries and conflicts they experience within themselves, conflicts with their close other or social network, and the kind of support they benefit from in the period from the patients’ diagnosis onward.

## Methods

### Study design

We conducted a qualitative interview study and report according to the Standards for Reporting Qualitative Research (O’Brien et al., 2014). The Medical Ethical Committee of the University Medical Center Groningen provided a waiver for the study not falling under the Medical Research Involving Human Subjects Act. The Ethics Committee of the Faculty of Behavioral and Social Sciences at the University of Groningen approved the study protocol (17075-O).

### Participants and procedure

A non-profit organization in Groningen, the Netherlands that advocates for those who receive or provide care approached their ICG platform and network of care professionals in palliative care via mail with the invitation for current and bereaved primary caregivers of persons with an incurable somatic illness (excluding dementia) to participate in an interview study. The platform and network were also asked to forward this invitation to people in their network (snowball sampling), and the organization advertised the invitation in their news-letter, social media accounts and on their website. Our research team sent the invitation flyer to organizations in the Netherlands representing informal caregivers or patient groups, and via flyers and social media posts on Facebook. We also recruited caregivers indirectly by reaching out to care professionals working with patients in the palliative phase of life, such as professionals working in oncology or at the unit for lung diseases. Interested caregivers contacted the first author using the provided contact details or gave care professionals permission to share their contact details with the first author. The first author phoned all interested caregivers and screened them for the eligibility criteria, relationship to the patient and informed them on more details of the study procedure. Eligibility criteria were both the ICG and patient being 18 years or older, having provided care to or currently caring for a close other during the last phase of their life, the care being unpaid for and informal, and in case of bereavement having provided care not too long ago (we discussed the time since bereavement together with the ICG to assess is recall bias would be an issue). Thereafter, caregivers received an information letter per email. All of them were willing to participate and signed an informed consent form on paper or digitally, before the interview took place.

### Data collection

The first author conducted semi-structured face-to-face interviews at a location of the ICGs choice where they felt comfortable and could speak freely without the care recipient being close by. We aimed to achieve triangulation in our data by inviting both current and bereaved ICGs as bereaved ICGs potentially have more mental capacity to reflect on their experiences and support needs during their caregiving period than those who were currently providing care. We developed the interview guide in collaboration with an internist-oncologist specialized in palliative care, a research and project coordinator for a regional non-profit organization that advocates for those who receive or provide care, and a panel of current and bereaved informal caregivers (see Supplemental Appendix A). During the interviews, we emphasized that we were focusing on the period from the close other’s diagnosis or the point that they received the message that there was no curative intent in the treatment anymore, to the present time (current ICGs) or to the passing of the close other (bereaved ICGs). All interviews were audio-recorded and took place between November 2017 and July 2018, and lasted between 40 and 92 minutes.

### Data analysis

The recordings were transcribed verbatim by students and anonymized. Accuracy of transcription was checked by listening to random samples from the recordings and checking the audio with the transcript. The transcripts were coded using reflexive thematic analysis (Braun and Clarke, 2006, 2012). In the first phase, the first author (AL) and a second coder (MT; all experienced in qualitative research) read the interviews carefully. The text fragments that represented specific content were marked on paper (phase 2). The two coders compared their coded fragments and in case of disagreement, discussed this until they reached agreement, and in case of continuing disagreement asked the opinion of a third coder. Based on the codes, a coding scheme was developed. The coding scheme was adapted to the codes emerging from new interviews by an iterative process. No new interviews were conducted as soon as data saturation was achieved. The marked text fragments and their related code have been uploaded in the program ATLAS.ti. Hereafter, as in line with phase 3 of Braun and Clarke (2006), we categorized all codes in subthemes. Next, we organized these subthemes according to the overarching themes of the current study, that is, to explore the wishes of ICGs, the conflicts they encounter(ed), what they found supportive, and what could make caregiving easier in this period. Finally, we reviewed the themes and subthemes with all coders (phase 4) and described the themes using quotes of participants (phase 5).

## Results

In total, we interviewed five current caregivers and fifteen bereaved caregivers (for their characteristics see Table 1); the time since the close other passed away ranged from 1 month until 10 years. The majority of caregivers were female (16 out of 20), and most of the ICGs took care of their partner (12) or a parent (7). The illness of the care recipient varied.

**Table 1.** Characteristics of the informal caregivers.

ICG	Current or bereaved ICG	Gender	Age	Close other	Disease of close other	Disease duration^a^	Passed away since
ICG1	Bereaved	Female	71	Partner	Aftermath of a stroke	3 years	Unknown
ICG2	Bereaved	Male	75	Partner	Cervical cancer	2 years	10 years
ICG3	Bereaved	Female	57	Parent	Liver cancer	1 month	1 month
ICG4	Bereaved	Male	43	Partner	Brain cancer	1 year	3 months
ICG5	Current	Female	63	Partner	Esophageal cancer	3 years, 6 months	NA
ICG6	Bereaved	Female	50	Partner	Lung cancer	1 year, 2 months	1 year, 7 months
ICG7	Bereaved	Female	36	Partner	Skin cancer	7 months	1 year, 7 months
ICG8	Current	Female	53	Partner	Neuroendocrine cancer	7 years	NA
ICG9	Bereaved	Female	37	Parent	Mesothelioma cancer	5 months	1 year, 1 month
ICG10	Bereaved	Female	51	Partner	Colon cancer	1 year, 7 months	1 year, 3 months
ICG11	Bereaved	Female	50	Partner	ALS	2 years	8 months
ICG12	Bereaved	Female	70	Sibling	Esophageal cancer	1 year	2 years
ICG13	Bereaved	Female	44	Partner	Lymph node cancer	7 years	1 year, 5 months
ICG14	Current	Male	55	Ex-partner	ALS	8 years	NA
ICG15	Current	Female	72	Partner	COPD	22 years	NA
ICG16	Bereaved	Female	47	Parent	Pancreatic cancer	2 months	2 months
ICG17	Bereaved	Female	57	Parent	Diabetes; bone infection	Unknown	9 months
ICG18	Bereaved	Male	34	Parent	MSA	3 years	3 years
ICG19	Bereaved	Female	25	Parent	Cancer	1 year, 4 months	2 months
ICG20	Current	Female	35	Parent	Kidney failure	13 years	NA

ALS (amyotrofische laterale sclerose) and MSA (Multiple system atrophy) are progressive neurodegenerative disorders.

COPD: chronic obstructive pulmonary disease; NA: not applicable.

a The disease duration was unknown for one person. The disease duration is the time since diagnosis; in some cases, it was known from the diagnosis onward that the disease was life-threatening and incurable, in other cases this became clear over time.

While coding the interviews, many quotes reflected on the unique palliative context, which will be described first. Advises that caregivers would have given themselves or other caregivers, often corresponded to subthemes (e.g. limit social contacts) and are therefore integrated in these sections. All subthemes and representing quotes are presented in Table 2.

**Table 2.** Overview of themes and subthemes representing informal caregivers wishes, conflicts, and support needs.

Theme	Subthemes	Quotes
Wishes of ICGs	Live life as normal as possible	ICG2 (Bereaved, male, 75): “Just the regular stuff, things normal families do. It should not be the case that there is a big dark cancer cloud, uh, hovering over this house.” ICG7 (Bereaved, female, 36): “Actually, from the beginning we have said that we will keep doing the things we used to do. We will not do any crazy stuff. I have asked ‘do you have a bucket list’… yes, but I don’t like it because you are doing it for the reason of… well yes, now the enthusiasm is gone, so we haven’t done it.”
	Quality time with close other and family	ICG9 (Bereaved, female, 37): “Having a lovely barbecue with all of us and just a couple of those small things is, yeah, that brings the silver lining, so to say. I was very aware of that. Enjoying everything a bit more, and if I had the afternoon off, taking my kids to [their] grandpa.”
	Create a comfortable life for close other	ICG2 (Bereaved, male, 75): “…that was making sure that she lacked nothing, I would almost say” ICG16 (Bereaved, female, 47): “I just wanted… ehm … that everything just went well for her. That nothing went wrong, that that… Yes, that she would be happy with everything all of the time, was satisfied with it. Because I was like, these are her final weeks/months and I just wanted that to go well.”
	Keep a life of your own, i.e. work, hobbies, social contacts	ICG5 (current, female, 63): “I don’t just want to provide care all the time. I just can’t keep that up. […] Of course I also want to have a good time with [name of partner], but I also want my own things.” ICG6 (Bereaved, female, 50): “…when I could go to the office for a few hours, and that truly was a blessing, because then I was in a normal environment for a while. And I said to my team, ‘don’t spare me’, you know, burn me down, or go on a rant, or, but don’t go to me ‘oh well, and let’s just not bother her’, no, because then I wouldn’t become a happy person, I just want to play along fully. So I loved that.”
Conflicts within caregivers and with others and experienced boundaries	ICGs wants to guard role of close other (e.g. partner, child) instead of becoming a nurse	ICG17 (Bereaved, female, 57): “So I do feel that I have been pushed into a role, no longer as a daughter and as a child, but I think: I have done things that I did not want to do as a child or daughter of my parents.” ICG11 (Bereaved, female, 50): “And that was something of which I said, uh, I want to remain your wife, I’m not going to do that. But I did. Until the 24-hour home care came. And then I said, uh, now I really don’t do it anymore. No.”
	Caregiving conflicts with ICG’s other activities	ICG19 (Bereaved, female, 25): “My daughter. She also needs caring, parenting. Taking her to school, to daycare… to swimming lessons. That takes a lot of time. I had school, I had an internship then… and I had my job. And besides… yeah… there wasn’t much time left to do something for myself or anything. But every now and then I went out to clear my head.” ICG9 (Bereaved, female, 37): “But, um, your social contacts, eh, they are completely, yes… are on the back burner. Still. Yes, because when you come home, you are also exhausted and then you really only want one thing: sleep.” ICG12 (Bereaved, female, 70): “I have a large garden behind, and in front and next to the house. Uh, I really liked that [gardening]. I still like it very much. And later I also noticed… that I thought “I haven’t done anything to that garden here for a damn year, it doesn’t look good either.”
	Limit the social contacts to those important to the patient and ICG	ICG4 (Bereaved, male, 43): “We have a fairly large network, many friends, acquaintances, et cetera. And there are many people who want to stop by for the sake of their own feelings. Well, we didn’t feel like that. We both didn’t.” ICG7 (Bereaved, female, 36): “Yes, it must be, for the children too, otherwise they will be hearing the same story all day. Especially the eldest made that clear at one point, ‘Ugh, visitors again.’ Yes, you also have to protect them from that, that they too…, their life also just goes on. When they come home, that they don’t mind coming home, because then ‘there will be people again and I have to hear the story again’… yes, a bit of protection.”
	Conflicts in wish for professional and/or informal support ICG and close other	ICG20 (current, female, 35): “I say: ’Did the home care come by? ‘No, I told them not to come.’ Then we have those fights again: ‘yes, but that’s pathetic, isn’t it?’ I say: ‘No that’s not pathetic, it’s their job, you know.’ Then yes, you have those kinds of conversations and afterward, I’m on the phone with home care” ICG9 (Bereaved, female, 37): “He didn’t want help at night and I wanted help at night. I said: ‘this is no longer possible.’ It’s really not possible anymore, because he sometimes got up from the bed, for example, and he was actually so weak that he couldn’t do that anymore. So he fell twice too, so I said… and he was really clear: ‘no, I don’t want any help.’” ICG8 (current, female, 53): “I don’t have a housekeeper or a cleaning lady either, because I, uh… He’s always at home. So if someone also comes in and starts cleaning… He hates that. […] Yes, it will certainly help me. I’m also sure that when he’s gone, I’ll get a cleaning lady right away (laughs).”
	Experienced boundaries	ICG17 (Bereaved, female, 57): “So I was available for him [parent] 24-7. I haven’t been able to set my own boundaries, but I kept thinking: ‘Oh, it’s only for a short while.’” ICG9 (Bereaved, female, 37): “I have two young children now and I cannot be there for my father day and night. As much as I want to, but I can’t. I have a family here too”
Helpful support	Environment offers practical support (i.e. doing groceries, taking over the care for the close other)	ICG6 (Bereaved, female, 50): “Drawing up a whole schedule of family members who wanted to help. And in the beginning there were also aunts, so my father’s sisters, who came on Wednesdays, for example, to do the household and provide care to him. Food and stuff. At those moments, I consciously did not visit, and at a certain point I asked: ‘yes, who wants to help? Who wants which day?’ Just made up a whole schedule.” ICG7 (Bereaved, female, 36): “At one point they [close friends] took turns, that there was always someone with me, they didn’t want me to be alone with him [father] anymore. Well, she [a close friend] would come one morning, and every once in a while she would run the vacuum cleaner through the house. ‘Well’, she says, ‘sit down for a while’, or with birthdays, that she arranged presents for me, just simple things, but that they were there.”
	Environment emotionally supports or acknowledges role of ICGs	ICG6 (Bereaved, female, 50): “Knowing that you had a kind of safety net in the background, and what pulled me through were the friends and family who continuously said, ‘we are here, you just have to say the word and then we are here’, that was very nice.” ICG9 (Bereaved, female, 37): “..that I could just express my frustration, my fears, my sadness with her [close friend], uh, without her passing a judgment on it. And also just said: ‘yes, it’s a lot. Come here for a moment’, put an arm around me and yes, the comfort.”
	Availability of professional care	ICG7 (Bereaved, female, 36) “.. the support of the general practitioner, especially in the last three weeks [of the patient’s life], he [GP] came by three, four times a week, and before that almost weekly, every 14 days, or he gave us a call. That was very supportive.”
Wished support	An overview of all support options	ICG3 (Bereaved, female, 57): “And I would have liked a little more guidance in that. And that, yes, so the education about that sort of thing, what is it really, an alarm. What is it anyway, X [name of home care organization]. How does it work, arranging home care, what is that… well things like that.”
	One point of contact for all practical regulatory matters	ICG13 (Bereaved, female, 44): “Well, it’s important at the moment that, that, one of the partners or children gets sick, so to speak, that they can go to one counter. That you can register there and at that moment, [the counter] do or arrange matters and, well, and then also offer the emotional support, so to speak.”
	An environment that pays attention to the wellbeing and upcoming loss of the ICG	ICG6 (Bereaved, female, 50): “Don’t just look at the patient, although he is very important. But that patient, whether he is terminally ill or not, that patient fares better if he has a partner or informal caregiver who also receives the necessary attention. Because the caregiver becomes extra motivated when she knows that she can tell her story. It is an interaction. So try to address the caregiver as well and do not focus solely on the care of the patient, because the partner suffers just as much, and that is sometimes… that is sometimes forgotten.”

### The palliative context

Many ICGs expressed that caring for their close other is out of love and self-evident, although a few ICGs experienced that the social or professional environment pushed them into the role of primary informal caregiver. One of the difficulties that ICGs experienced in their situation is the uncertain and unpredictable character of the illness process. Stable periods can suddenly be interrupted by worsening of the disease or disease-related symptoms. In addition, one ICG (ICG8, female, 53) explained that she feels that her life is on hold, waiting for the devastating loss of her close other. ICGs described that their burden increases over time, being intensive toward the near end of the close other. It varied whether patients or ICGs wished to know about the estimated time given until the patient’s death. It also varied whether ICGs and patients preferred to discuss the upcoming death of the patient, and whether they discussed with the close other what their life would be like after the close other’s death. One ICG (ICG14, male, 55) advised caregivers to discuss with their close other which obstacles can be expected in the course of the disease, and which choices both want to make in this. He experienced that not discussing this made it difficult to respond to certain situations at the moment his close other became incapacitated.

### Wishes of informal caregivers

#### Live life as normal as possible, spend time together, and comfort the close other

ICGs stressed the importance to continue their life as normal as possible after receiving the news of the disease being incurable, especially when children were involved. ICGs indicated that spending quality time with the close other and the family was important, and some ICGs described that especially the simple things in life make it count. Making sure that the close other felt comfortable was also expressed as a priority for ICGs.

#### Keep a life of your own

ICGs felt that it was important to “keep a life of your own,” which could entail that ICGs continued their work, their social contacts or their hobbies. It was considered important, or even necessary, for ICGs to attend environments in which they could be themselves without the focus on their role as caregiver. This was reflected in the advice ICGs gave to “guard your own life,” keep doing the things that recharge your battery.

### Conflicts within caregivers and with others and experienced boundaries

#### Conflicts within caregivers: guard the role

Several ICGs indicated that they felt uncomfortable with the changes in their role as caregiver and related tasks, experiencing conflicts within themselves. Some partners stated that they wanted to stay the romantic close other instead of becoming a nurse for their partner. ICGs caring for a parent experienced difficulties with certain care tasks, mainly the personal care tasks like assisting with showering or going to the toilet, since it crossed intimacy barriers.

#### Conflicts within caregivers: Balance caregiving and other activities

Being an informal caregiver often conflicted with other activities that ICGs used to spend their time and energy on, such as work, their social life, and hobbies. Some caregivers experienced continued tension between their wish to be a good caregiver and their wish to maintain their other activities. Other caregivers chose to dedicate their time solely to the caregiving and withheld or paused their other activities for a certain period. Especially ICGs having a household of their own and/or having children indicated that they felt that it was important to focus on their own household and therefore limit their availability toward the ill close other.

#### Conflicts with others: limit social contacts

Some ICGs did not appreciate or refused the social visits from acquaintances with whom they had little contact and who only wanted to visit their close other “to say goodbye for their own sake.” One ICG (ICG5, female, 63) explained it as “they just came over to gawk.” ICGs also noticed that the close other kept up a good appearance during the social visit, which could be very intensive. As a consequence, ICGs had to deal with a tired and moody close other afterward. ICGs advised to make sure to balance the time you and your close other spend with social contacts, and the quality time that you spend together.

#### Conflicts with others: conflicting wishes for additional support

A situation in which ICGs experienced conflicting wishes between themselves and the close other was when the ICG wished to have (more) professional or informal support than the close other. In some cases the close other wished to be taken care of only by the ICG because they believed that the ICG is the only person who can provide care to them with enough time and attention, or they felt uncomfortable to be taken care of by others in personal caregiving tasks, thereby asking more support than the ICG was able or willing to provide. In other cases, the caregiver was reluctant to introduce more persons to the household to provide informal or professional support as this would intrude on their privacy.

#### Experienced boundaries

ICGs explained that their boundaries changed over time, and that they had performed tasks that they previously could not have imagined doing. Some ICGs wanted to do everything for their close others and did not set any boundaries, whereas others were aware of their boundaries and felt that they managed to act accordingly. Some ICGs indicated that they did not know or were unaware of their boundaries. A part of the bereaved ICGs mentioned that they noticed in hindsight that they had crossed several of their boundaries even though they were often unaware of this when it actually happened.

### Helpful support

#### Practical support

ICGs indicated that practical support, such as helping in the household, doing groceries or taking over the care of the close other so that the caregiver could do something for himself or herself inside or outside the house, was considered helpful.

#### Emotional support

ICGs were appreciative of persons in their environment with whom they could talk about their personal experiences. The feeling of being supported by persons in the environment, and feeling acknowledged for being an individual who is going to lose his or her close other, was experienced as helpful.

#### Professional support

The availability of good professional support was also considered of value, for example regular calls or visits from the general practitioner.

### Wished support

#### Insight in support options

ICGs indicated that they would like to have an overview of all the support options that are available to them, and where and how they can apply for this support. This applies to practical support tools, such as an alarm or a special bed, as well as for professional care such as getting help from a night nurse or a home care organization.

#### One point of contact

ICGs expressed that it would be helpful if there was one contact person or contact point that would help arrange or take over all the administrative and logistic issues related to the caregiving, such as applying for financial aids or practical support tools (e.g. an alarm, special bed).

#### An environment that guards ICG’s wellbeing

Some ICGs indicated that they experienced difficulties in recognizing when the situation became too much for them to handle. ICGs would benefit from a professional and/or private environment that pays attention to the wellbeing of the ICG, and warns the ICGs in due time when they signal that the ICG is overburdened.

## Discussion

Providing care to a close other who is diagnosed with an incurable, life-threatening illness can be described as an act of love, is sometimes self-evident and often a rollercoaster all together. When faced with a devastating diagnosis of a close other, caregivers did not mention exciting bucket lists, but often wished to continue life as normal as possible while providing good care to their close other. This has been reported differently for several caregivers for whom the ALS diagnosis of their close other led to drastic life changes (Iseli et al., 2024). Caregivers emphasized the importance of “keeping a life of your own,” especially when they were seen as themselves and not as a caregiver during these activities or in these contexts. This is in line with caregivers of patients with a brain tumor who stressed that their work made them feel important and useful again, and offered a temporary escape from the illness situation (Dahlberg et al., 2022). Caregivers of a close other with dementia reported the necessity of designated personal time to balance caregiving with self-care to recharge their energy levels (Boots et al., 2015) and the importance of family support to provide this personal time (Lindeza et al., 2020), which was also stressed in our study. However, even though some caregivers in our study were aware of their need to guard their own activities, they experienced conflicts in their wish to pursue these activities and their wish to provide good care to their close other. Another conflict ICGs experienced was that ICGs wished to receive more support from the personal or professional network to make things easier for them, while the close other wished to preserve their privacy or to be cared for by the ICG. These struggles have been reported before and often ICGs choose to prioritize the needs of the close other over their own (de Wit et al., 2019; Grande et al., 1997; Zapart et al., 2007). This might be somewhat sustainable in case of short-term illness trajectories, but when the illness progresses over a longer period, caregivers may not be able to continue caregiving (Shyu, 2000).

Caregivers wished for more practical support such as an overview of available support options and how to apply for these (Zapart et al., 2007) and one contact point that helps limiting caregivers’ administrative and logistic burden, which has been reported before by ALS caregivers (de Wit et al., 2019). In the Netherlands, the practical support is organized by the health care system (e.g. availability of good professional care such as home care or social work, finances covered by health care insurances) and by legal regulations (e.g. paid caregiving leave for working caregivers). Several municipalities even provide professionals that advise ICGs on or take over logistic and administrative issues related to caregiving free of charge. Apparently caregivers did not know about the existence of and how to access these services, showing a mismatch between the supply and the demand of services, which has been reported before by caregivers providing care to a close other with dementia (van der Roest et al., 2009). Caregivers considered it helpful to be emotionally supported (also reported by Harding et al. (2012)), but wished for an environment that guards their wellbeing, perhaps to compensate for their personal difficulty to preserve their wellbeing.

Not being recognized as caregiver by themselves and by care professionals (Peters et al., 2020) may also withhold ICGs to apply for resources or (financial) support. Some ICGs reported to “just care for their close other” and only found out that they were considered a caregiver at a late stage or even after the passing of their close other. Even if professionals identify and reach out to support caregivers, caregivers may not accept the offered support. Looking in hindsight, some bereaved caregivers explained that they would have wanted to talk to a professional, although they also stress that they probably would not have accepted this support at that time, given their limited time and energy. This paradox has been reported before in caregivers of metastatic cancer (Mangan et al., 2003) and in dementia caregivers; later-stage dementia caregivers retrospectively acknowledged their wish to talk to someone in the early stages of the disease, whereas early-stage dementia caregivers did not recognize this need for support (Boots et al., 2015). Also, some ICGs in our study indicated not being aware of their own boundaries in providing care, which would further complicate accepting support. This makes it extremely challenging to offer ICGs the right support at the right time, as this would entail to convince ICGs to accept support in a hectic period with the argument that it would probably help them in the long run. In addition, what professionals consider to be the right time to start palliative care, does also differ between countries (Giannouli et al., 2019).

Reflecting on the findings of this study, we provide some implications to better support caregivers in the palliative phase. First, our advice would be that the professionals involved in the patient’s care inform the people surrounding the patient that they are considered caregivers, and direct them to caregiver organizations or information about available professional and informal support, legal regulations (e.g. paid caregiving leave for working caregivers), and insurances. Secondly, caregivers should be convinced of the importance of guarding their own mental and physical wellbeing to support the close other in the long run, and caregivers are advised to ask people in their environment to keep an eye on their wellbeing. Thirdly, to maintain caregivers’ wellbeing, caregivers need to balance one’s own needs with the requests that come with caregiving, which requires caregivers to become aware of their boundaries, permit themselves to take their boundaries seriously, and have the tools to communicate or act upon their boundaries. Caregivers should be guided in structurally reflecting on their needs and boundaries as they forget to do so in the daily hassle, and also since needs and boundaries may change over the illness course. Formal carers can play an important role in these steps, although, given the increasing burden on formal care, digital tools can also support caregivers in this process. We have developed such a tool for Dutch-speaking caregivers. On a national level, campaigns may emphasize the emotional impact of being a caregiver and provide tips for the personal environment how to support caregivers both practically and emotionally.

### Strengths and limitations

A strength of this study is that it addressed conflicts experienced by ICGs within themselves and with others, which are important contributors to daily stress, in order to offer directions to support ICGs. Moreover, by including both current and bereaved ICGs, and by including various diagnoses the care recipient faced, the caregiving experiences are described from multiple perspectives.

Unfortunately, we did not reach a balanced sample of current and bereaved ICGs, as it was difficult to recruit current ICGs. Most bereaved caregivers had lost their close other 1–3 years ago, although one caregiver had lost his close other 10 years ago. Bereaved ICGs reflect on their experiences with more emotional distance, although this also yields the possibility of recall bias. A previous study described a trend that bereaved caregivers discussed more positive points of caregiving, although bereaved and active caregivers overall stated similar priorities for end-of-life care (Mangan et al., 2003). Including more current ICGs, may have also resulted in some additional codes or themes. The majority of ICGs were of female gender, which corresponds with gender distributions in Europe (Elayan et al., 2024), although a meta-analysis shows that gender differences were small to very small with respect to caregiver stressors, social resources, and caregivers’ health (Pinquart and Sörensen, 2006). ICGs in this study provide care to persons with a variety of illnesses, still 13 out of 20 patients were diagnosed with cancer. The caregiving experience may differ depending on the influence the close other’s illness has on their body, cognition and mental wellbeing, for example altering personality, or cognitive functioning (Lipsman et al., 2007). It was found before that the views of caregivers on the functioning of their close other, together with their own illness perceptions, differ, and could even be biased, which in turn could influence how they perceive the necessity to provide care and in turn how burdened they are by having to provide this care (Giannouli and Tsolaki, 2022; Zarzycki et al., 2023; Zarzycki and Morrison, 2021). Also, the life expectancy of patients might have varied. One can imagine that providing care to a close other whose life expectancy is measured in months may result in a different experience with respect to the emotional impact, the tasks and the intensity of care to provide as compared to when the close other’s life expectancy is estimated in years, and caregivers slowly grow in their caregiving role but also may gradually become exhausted. A meta-analysis on caregiver burden showed that the type of diagnosis did not, but the level of anxiety of caregivers did influence the level of experienced burden, suggesting that the type of illness might not be directly related to caregiving burden, but the anxiety it provokes in the caregiver is (Del-Pino-Casado et al., 2021). It is therefore important to keep in mind that caregivers who are more anxious might be in need of care and support most, regardless of the medical situation their close one is in. Moreover, all interviewed ICGs were users of the Dutch health care system and support options available in the Netherlands, which may vary from the available support in other countries.

### Conclusion

Many caregivers experienced their role as an act of love and saw it as self-evident, even though a balance between caregiving and their own wishes was sometimes hard to find. An overview of possible care options as well as guidance in balancing their own wishes and boundaries with the requests of caregiving could be helpful to decrease conflicts.

## Supplemental Material

sj-docx-1-hpq-10.1177_13591053251357769 – Supplemental material for Wishes, conflicts, and support needs of informal caregivers of patients in the palliative phase: A qualitative study Supplemental material, sj-docx-1-hpq-10.1177_13591053251357769 for Wishes, conflicts, and support needs of informal caregivers of patients in the palliative phase: A qualitative study by Anne Looijmans, Marrit Annika Tuinman, Marieke Nanninga and Mariët Hagedoorn in Journal of Health Psychology

sj-jpg-2-hpq-10.1177_13591053251357769 – Supplemental material for Wishes, conflicts, and support needs of informal caregivers of patients in the palliative phase: A qualitative study Supplemental material, sj-jpg-2-hpq-10.1177_13591053251357769 for Wishes, conflicts, and support needs of informal caregivers of patients in the palliative phase: A qualitative study by Anne Looijmans, Marrit Annika Tuinman, Marieke Nanninga and Mariët Hagedoorn in Journal of Health Psychology
