# Supplementary material for: Wishes, conflicts, and support needs of informal caregivers of patients in the palliative phase: A qualitative study
Source: J Health Psychol. 2025 Aug 7;31(4):1662–76. doi: 10.1177/13591053251357769 (PMC12960756; doi:10.1177/13591053251357769)
Supplement: sj-docx-1-hpq-10.1177_13591053251357769 – Supplemental material for Wishes, conflicts, and support needs of informal caregivers of patients in the palliative phase: A qualitative study [file sj-docx-1-hpq-10.1177_13591053251357769.docx]

**Figure 1.**

***Participant recruitment, eligibility, and retention for study completion***


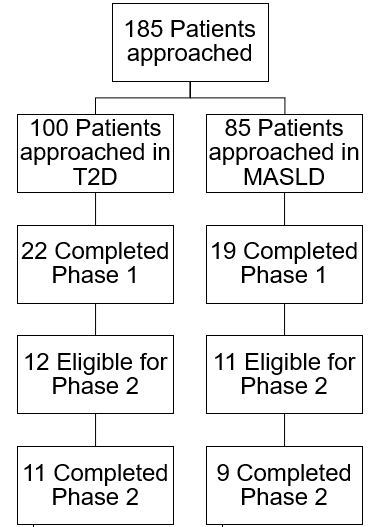


Figure legend. T2D=Type 2 Diabetes; MASLD=Metabolic Dysfunction-Associated

Steatotic Liver Disease.
