# Supplementary figures and images for: Wishes, conflicts, and support needs of informal caregivers of patients in the palliative phase: A qualitative study
Source: J Health Psychol. 2025 Aug 7;31(4):1662–76. doi: 10.1177/13591053251357769 (PMC12960756; doi:10.1177/13591053251357769)

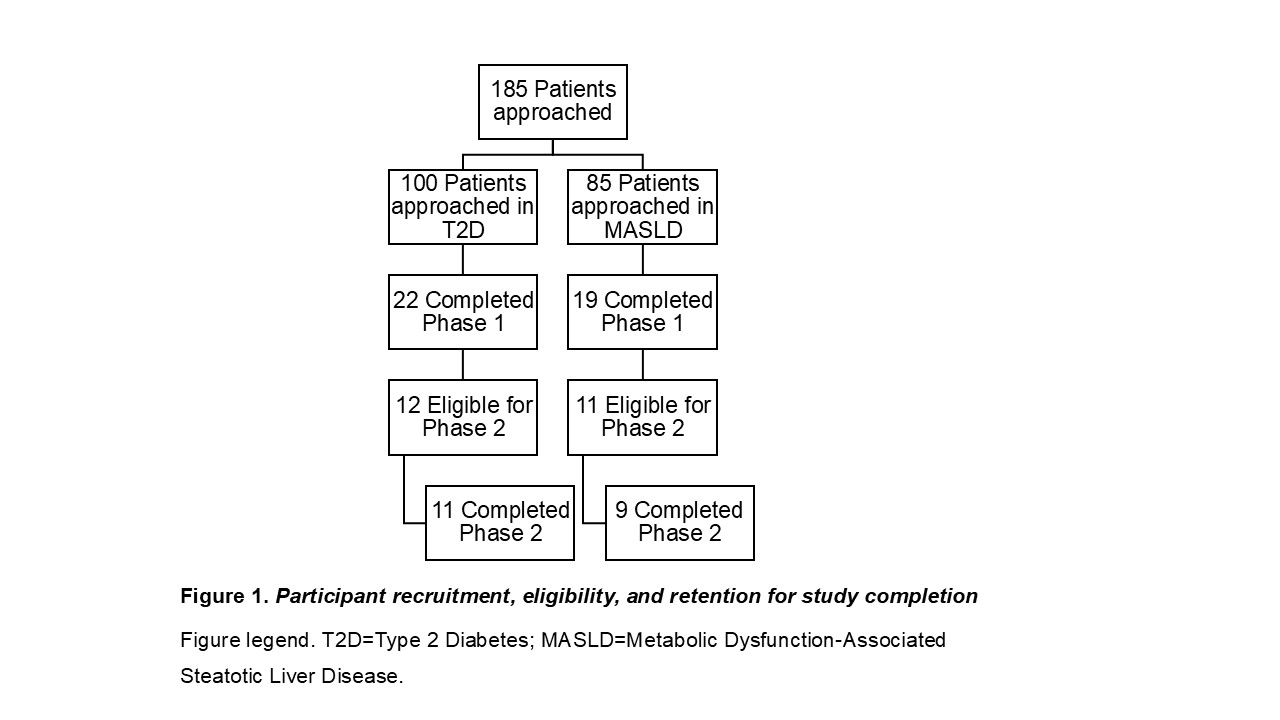

Supplement: sj-jpg-2-hpq-10.1177_13591053251357769 – Supplemental material for Wishes, conflicts, and support needs of informal caregivers of patients in the palliative phase: A qualitative study [file sj-jpg-2-hpq-10.1177_13591053251357769.jpg]
